# Compositional Structure of the Genome: A Review

**DOI:** 10.3390/biology12060849

**Published:** 2023-06-13

**Authors:** Pedro Bernaola-Galván, Pedro Carpena, Cristina Gómez-Martín, Jose L. Oliver

**Affiliations:** 1 Department of Applied Physics II and Institute Carlos I for Theoretical and Computational Physics, University of Málaga, 29071 Málaga, Spain; rick@uma.es (P.B.-G.); pcarpena@ctima.uma.es (P.C.); 2 Department of Pathology, Cancer Center Amsterdam, Amsterdam UMC, Vrije Universiteit Amsterdam, 1081 HV Amsterdam, The Netherlands; c.a.gomezmartin@amsterdamumc.nl; 3 Department of Genetics, Faculty of Sciences, 18071 and Laboratory of Bioinformatics, Institute of Biotechnology, Center of Biomedical Research, University of Granada, 18100 Granada, Spain

**Keywords:** DNA compositional structure, sequence compositional complexity, segment compositional signature, hierarchical genome structure, evolutionary adaptive trends

## Abstract

**Simple Summary:**

DNA structural biology deals with the understanding of DNA and three-dimensional chromatin structure, which can determine its function in the cell. The key structural properties of the DNA fiber, such as stability, flexibility, and susceptibility to damage, largely rely on the composition of the DNA sequence. Variations in the nucleotide sequence result in a patchy chromosome structure, which is formed due to the differential GC content of exons, introns, regulatory elements, repeats, etc. The compositional structure of a genome at different length scales may be revealed via the use of entropic segmentation algorithms or fluctuation analysis of DNA walks. The former algorithms divide the four-symbol nucleotide sequence, or its two-symbol variants, into an array of compositionally homogeneous, non-overlapping domains, isochores, and compositional superstructures, all of which are hierarchically organized in the chromosome. Once the compositional structure of a genome is known, the compositional genome signature or sequence compositional complexity (SCC) can be computed, enabling the comparison of genome structures.

**Abstract:**

As the genome carries the historical information of a species’ biotic and environmental interactions, analyzing changes in genome structure over time by using powerful statistical physics methods (such as entropic segmentation algorithms, fluctuation analysis in DNA walks, or measures of compositional complexity) provides valuable insights into genome evolution. Nucleotide frequencies tend to vary along the DNA chain, resulting in a hierarchically patchy chromosome structure with heterogeneities at different length scales that range from a few nucleotides to tens of millions of them. Fluctuation analysis reveals that these compositional structures can be classified into three main categories: (1) short-range heterogeneities (below a few kilobase pairs (Kbp)) primarily attributed to the alternation of coding and noncoding regions, interspersed or tandem repeats densities, etc.; (2) isochores, spanning tens to hundreds of tens of Kbp; and (3) superstructures, reaching sizes of tens of megabase pairs (Mbp) or even larger. The obtained isochore and superstructure coordinates in the first complete T2T human sequence are now shared in a public database. In this way, interested researchers can use T2T isochore data, as well as the annotations for different genome elements, to check a specific hypothesis about genome structure. Similarly to other levels of biological organization, a hierarchical compositional structure is prevalent in the genome. Once the compositional structure of a genome is identified, various measures can be derived to quantify the heterogeneity of such structure. The distribution of segment G+C content has recently been proposed as a new genome signature that proves to be useful for comparing complete genomes. Another meaningful measure is the sequence compositional complexity (SCC), which has been used for genome structure comparisons. Lastly, we review the recent genome comparisons in species of the ancient phylum Cyanobacteria, conducted by phylogenetic regression of SCC against time, which have revealed positive trends towards higher genome complexity. These findings provide the first evidence for a driven progressive evolution of genome compositional structure.

## 1. Introduction

DNA structural biology focuses on the understanding of the three-dimensional structure of DNA, which plays a vital role in determining its function in the cell. Key structural properties of the DNA fiber, such as its stability, flexibility, or susceptibility to damage, largely depend on the composition of the DNA sequence (i.e., the specific arrangement of nucleotides within the DNA sequence). Notably, regions of DNA that exhibit a high proportion of guanine and cytosine (known as GC-rich regions) tend to possess a more stable structure due to the stronger hydrogen bonding that occurs between these nucleotides. The presence of histones or histone-like proteins in the genome can also have a significant impact on genome structure organization and long-range genome interactions. Recent advances have enabled chart maps of histone modifications and related chromatin structures. These maps provide insights into the intricate relationship between chromatin and genome function, emerging in the understanding of large-scale domains and higher-ordered chromatin organization [1].

Since the genome encodes all historical information regarding a species’ biotic and environmental interactions, the analysis of genome compositional changes may provide important insights into the organization and evolution of genome structure over time [2,3,4]. Pioneering studies by Bernardi and collaborators [5,6,7,8] used the analytical ultracentrifugation of bulk DNA to uncover the observation that mammalian genomes are made up of isochores, which are long DNA segments of a typical size around 300 kb, and they are fairly homogeneous in G+C content. Nucleotide composition has been related to important genome features such as the so-called genomic code [9], the 3-D structure of DNA [10], or the existence of topologically associating domains (TADs) in the genome [11,12]. The recent achievement of the first complete 3.055 billion base pair sequence of a human genome [13] now provides an unprecedented opportunity to examine the isochore theory and study the evolution of DNA sequence structure. The application of statistical physics methods (such as power spectra, fluctuation analysis in DNA walks, or entropic segmentation) has facilitated the study of large-scale genome structures [14,15,16,17], revealing the presence of long-range, power law correlations in DNA sequences and suggesting a fractal (scale-invariant) structure of the genome. However, such a genome landscape directly contradicted the well-known characteristic lengths observed in most genome elements (genes, exons, introns, transposable elements, and so on). It was also particularly inconsistent with the isochore theory of the genome, which describes the genome as a mosaic of compositionally homogeneous segments known as isochores [2,6,18,19,20,21]. The paradox between a scale-invariant versus an isochore model for the genome was resolved by discovering that deviations from power law behavior can be observed in correlations [22] and that such deviations can be associated with isochore-like regions in, at that time, the best-sequenced eukaryotic genome assemblies [21,23]. Subsequent studies revealed that isochores are not the longest compositional domains in the genome as they are organized at even longer scales into compositional superstructures that are about two orders of magnitude longer than isochores [24]. By using a segmentation model that accounts for long-range correlations, these authors were able to determine a genomic map of the chromosome boundaries of superstructures based on rigorous statistical criteria.

Several measures of genome complexity are now available [25,26,27,28]; we refer the reader to [29] for a recent review. Some of these measures rely on the frequencies of *k*-words or *k*-mers. The complexity of the DNA sequence is computed at a length scale given by the word size (k), which maximizes the variability of word frequencies. However, given the presence of different length scales in the genome [15,21], choosing the right value for the parameter *k* is not an easy task.

An ideal measure of compositional structural complexity would consider the array of homogeneous domains of different lengths and compositions along the genome. Subsequently, it would calculate a complexity value based on statistical criteria. Sequence compositional complexity, or SCC [25], fulfills these criteria. This method first decomposes the nucleotide sequence into a number of homogeneous compositional domains under strict statistical criteria and then incorporates an entropic measure that accounts for the length and compositional differences among these domains. Recently, SCC has been employed to assess genome complexity in Cyanobacteria [3], providing the first evidence for the driven progressive evolution of genome compositional structure.

This review provides an overview of the theory and methods for DNA sequence segmentation (Section 2) and the modifications of the original segmentation algorithm, which can be used to find the largest genome compositional structures: isochores (Section 3) and superstructures (Section 4). Section 5 and Section 6 focus on the use of compositional segmentation to quantify the compositional structure of genomes. Specifically, we show that the distribution of segment G+C content has the desirable properties of a genome signature (Section 5), while in Section 6, we review sequence compositional complexity (SCC), a measure particularly convenient for quantifying the complexity of genome structure. In Section 7, we show the usefulness of SCC for uncovering phylogenetic trends in the ancient phylum Cyanobacteria, in which evidence for the driven progressive evolution of SCC was first found. Finally, in Section 8, we present conclusions.

## 2. DNA Sequence Segmentation

Given the pervasive spatial heterogeneity in nucleotide composition found in most genomes [30], the identification of compositional domains within a sequence is essential to understand genome structure [2]. As a result, this task holds significant importance in computational molecular biology [31], being the key step in understanding the spatial, large-scale structure of the genome. In simple DNA sequences that lack long-range correlations, such as those predominantly integrated by coding regions in prokaryotes, compositional patches can be easily identified [32]. However, for complex long-range correlated DNA sequences typical of eukaryotic genomes, the identification of homogeneous compositional domains becomes more challenging given the lack of a characteristic patch length [33,34]. To overcome this problem, a statistical approach capable of estimating, with a given level of confidence, the location of the boundaries separating compositional patches in a sequence should be used.

To divide a four-symbol DNA sequence into an array of compositionally homogeneous, non-overlapping domains, a heuristic, iterative segmentation algorithm [19,35,36,37] can be used. In brief, given a DNA sequence *S* of size *N,* a sliding cursor is moved along the sequence (*i =* 1, …, *N*), and the position, *i*, that optimizes a proper measure of compositional divergence between the left (*S*_1_) and right (*S*_2_) parts is selected. We choose the Jensen–Shannon divergence [36] as a divergence measure, as it can be directly applied to symbolic nucleotide sequences. The Jensen–Shannon divergence (*JS*) between two sequences, *S*_1_ and *S*_2_, is defined as follows:

(1) $$JS\left(S_{1},S_{2}\right)=H\left(S\right)-\left[\frac{n_{1}}{N}H\left(S_{1}\right)+\frac{n_{2}}{N}H\left(S_{2}\right)\right]$$

where *n*_1_ and *n*_2_ are the sizes of sequences *S*_1_ and *S*_2_, *S* is the sequence of size *N* = *n*_1_ *+ n*_2_ obtained by putting together *S*_1_ and *S*_2_, and *H*(·) is the Shannon entropy of the distribution of the relative frequencies of symbol occurrences:

(2) $$H\left(S\right)=-\sum_{i=A,T,C,G}^{}f_{i}\log_{2}f_{i}$$

If the divergence is statistically significant at a given significance level (e.g., s = 0.95), the sequence is split into two segments. Note that each pair of resulting segments is more homogeneous than the original sequence. The two resulting segments are then independently subjected to a new round of segmentation. The process continues iteratively over the new segments while sufficient significance continues appearing. Since Shannon entropy is invariant under symbol interchange, the segmentation algorithm and the SCC values derived from it are invariable relative to sequence orientation. Note that this segmentation algorithm can be easily generalized to accommodate other alphabets that are different from the standard four-letter one (A, T, C, and G) while preserving its properties. For instance, a twelve-letter alphabet (the four letters in the three codon positions) can be used to determine coding region borders [38]. In addition, if the sole interest lies in the compositional structure of G+C content, the algorithm can be adapted to a two-letter alphabet: Prior to segmentation, the four-letter DNA sequence is converted into a binary sequence with only two symbols comprising S (strong) when the nucleotide is C or G and W (weak) when the nucleotide is A or T. This mapping of the DNA sequence into a binary sequence is commonly known as strong/weak or simply the S/W rule. Specifically, this alphabet is used in the prediction of isochore boundaries (Section 3) and the search for compositional superstructures (Section 4.1).

The statistical significance level, *s*, represents the probability that the difference between adjacent domains is not solely due to statistical fluctuations, assuming the null hypothesis that the sequence is random and i.i.d. (independent and identically distributed). By adjusting the value of this parameter, one can obtain the underlying distribution of segment lengths and nucleotide compositions at different levels of detail [37], thus conveniently fulfilling one of the key requirements of a complexity measure [28]. Choosing a random i.i.d. sequence as the null hypothesis serves as a reference for homogeneity. In other words, a sequence is considered heterogeneous (and therefore, should be segmented) when differences in composition exceed what would be expected in a random i.i.d. sequence. Recent improvements to this segmentation algorithm [37] have enabled the segmentation of sequences with long-range correlations. The presence of correlations makes these sequences much more heterogeneous than random i.i.d. sequences; consequently, the method for computing significance level *s*, i.e., the reference for homogeneity, needs to be modified, as the algorithm may otherwise identify segments that appear trivially in the sequence due to the correlations. In such cases, the model adopted for homogeneous sequences is fractional Gaussian noise. Implementation details, source codes, and executable binaries for different operating systems can be downloaded from the following website: https://github.com/bioinfoUGR/segment (accessed on 20 April 2023) and https://github.com/bioinfoUGR/isofinder (accessed on 20 April 2023). 

In all cases, the result is the segmentation of the original sequence into an array of contiguous, non-overlapping segments (or compositional domains) for which their nucleotide composition is homogeneous at the chosen significance level, s.

## 3. Prediction of Isochore Boundaries at the Sequence Level

The genomes of warm-blooded vertebrates (such as mammals and birds) are made up of isochores, which are long DNA segments (~300 kb) that are fairly homogeneous in G+C content and that were first revealed by the analytical ultracentrifugation of bulk DNA [5,6,7,8]. The relevance of isochores is derived from the distinctive frequencies of genes, SINE (short interspersed repetitive elements), and recombination frequency, which are all higher in (G+C)-rich isochores, whereas LINEs (long interspersed repetitive elements) are denser in (G+C)-poor isochores [7]. Beyond compositional differences, the boundaries of isochore often correspond to chromosome regions that differ in replication timing, as observed in the isochores of the human major histocompatibility complex (MHC) locus [39], or in recombination rates, as observed in the human neurofibromatosis NF1 region [40]. Isochores can be found in a large variety of taxa, including unicellular eukaryotes [41], plants [42], and cold-blooded vertebrates [43], although they are more conspicuous in the genome of warm-blooded vertebrates (see [7] and references therein). The isochore theory has expanded our understanding of the complexity and compositional variability of eukaryotic genomes [44], and it is considered a fundamental level of genome organization [45,46]. The evolutionary origin and maintenance of isochores in present-day genomes has been subject to active debate [20,45,47,48,49,50].

The advent of large-scale DNA sequencing projects generating a substantial number of large DNA sequences [51] has led to the search for a direct test of the isochore theory. Our group developed an algorithm, based on the compositional segmentation described above, that is able to predict isochore boundaries at the sequence level [19,25,36,52]. Most large homogeneous genome regions predicted by this algorithm were identified with Bernardi’s isochores, showing correlations with biological features such as gene density, SINE and LINE (short, long interspersed repetitive elements) densities, recombination rate, and SNP (single nucleotide polymorphism) variability [52,53]; its accuracy as compared with other methods when applied to natural as well as simulated sequences has been proven [31,52,54,55]. Note however that if one chooses to conduct simulation experiments, a problem arises in which the sequences generated in the experiment have to be at least as complex as the natural ones, which is not an easy task because the sequences of higher organisms (mainly mammals and birds) usually have long-range power law correlations. In this way, a complete, reliable test for the isochore theory requires high-quality T2T genome sequences (see below).

The *IsoFinder* algorithm is designed to predict isochore boundaries [19,52,53], and it works as follows. Since we are interested in segments with defined G+C content, in the first place, the sequence to be analyzed is converted into a binary sequence using the S/W rule (Section 2). As we wish to detect only isochore-like DNA segments, we need to modify the original segmentation algorithm in order to avoid the influence of short-scale G+C heterogeneities on statistical significance. Thus, we filter nucleotide heterogeneities below a given minimum length, *L*_0_, and then compute the GC% content in left and right windows. In this way, we convert the subsequence of length *Lleft* (*Lright*) into an array of *Lleft*/*L*_0_ (*Lright*/*L*_0_) real numbers corresponding to the GC% content of each window of size *L*_0_. *IsoFinder* allows the user to choose among three different values of *L*_0_ (1, 2, and 3 kb) to perform the filtering procedure. It is advisable, however, to use *L*_0_ = 3 kb, which corresponds to a homogeneity criterion for mammalian isochores, and it is derived from the ultracentrifugation of DNA of different molecular weights [6].

**Figure 1:**
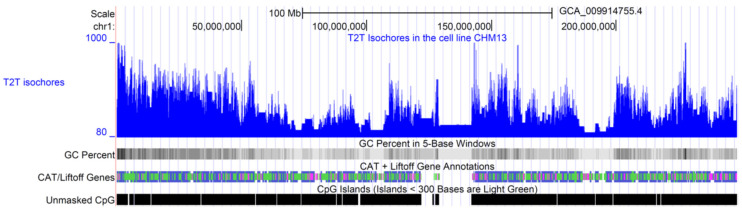
T2T human isochores. The image shows the isochore map of the T2T-CHM13 DNA sequence of human chromosome 1, obtained by plotting the isochores predicted by *IsoFinder* [19] with the help of the UCSC Genome Browser [56,57]. Blue line indicate the GC content of each isochore. The complete chromosome sequence was obtained by the Telomere-to-Telomere (*T2T*) Consortium [13], which includes gapless assemblies for all chromosomes except Y. The completed regions include all centromeric satellite arrays and recent segmental duplications. Tracks for G+C density in 5-base windows, genes, and CpG islands, taken from the UCSC Genome Browser database, are also plotted for comparison. The online isochore maps for all chromosomes are available at the UCSC Genome Browser: https://genome.ucsc.edu/s/oliver/T2T%20human%20isochores (accessed on 20 April 2023).

One of the main limiting factors relative to validating the isochore theory was the lack of high-quality DNA sequences. To overcome this problem, we applied *Isofinder* to the first complete 3.055 billion base pair T2T sequence of a human genome [13]. The obtained isochore and superstructure coordinates were then shared in a public database (https://genome.ucsc.edu/s/oliver/T2T%20human%20isochores (accessed on 20 April 2023)). Using PacBio HiFi and Oxford Nanopore, ultralong-read sequencing gapless assemblies were obtained for all chromosomes (except Y) of the homozygous CHM13hTERT cell line, which corrects errors in the prior references and introduces nearly 200 million base pairs of additional sequences [13]. Table 1 shows the lengths and GC% of T2T human isochores by chromosome, while Table 2 shows a summary statistic. The T2T human isochore map of chromosome 1, obtained with *IsoFinder* and plotted with the help of the UCSC Genome Browser [56,57], is shown in Figure 1. The online isochore maps for all chromosomes are available at the UCSC Genome Browser: https://genome.ucsc.edu/s/oliver/T2T%20human%20isochores (accessed on 20 April 2023). Note that besides the image of isochore maps for every chromosome, this website provides access to tables with specific genome coordinates for each isochore by using Table Browser (https://genome.ucsc.edu/cgi-bin/hgTables?hgsid=1627501187_vJA8raAFaxmpp4jAbRjEuzBiYujn (accessed on 20 April 2023)). In this way, interested researchers can now use T2T isochore data, as well as the annotations for different genome elements available on this website, to check a specific hypothesis about genome structure.

## 4. Long-Range Correlations and Compositional Superstructures in the Genome

The application of statistical physics methods to DNA sequences led to the discovery of long-range correlations, i.e., correlations between nucleotides over long distances along the DNA chain [15,16,17,33,58]. The stochastic properties of nucleotide sequences were studied by constructing a 1:1 map of the nucleotide sequence onto a walk (DNA walk). In this way, the mapping was used to provide a quantitative measure of the correlation between nucleotides over long distances along the DNA chain. Long-range power law correlations uncovered by these powerful methods imply a new scale-invariant property of DNA. However, from the point-of-view of genome structure, these long-range, power law fractal correlations also imply that compositional segments should appear at all scales (i.e., showing a power law distribution of segment lengths), a prediction that was confirmed by analyzing the length distributions of compositional domains resulting from sequence segmentation [36]. 

The emerging genome landscape of genome structure challenges the conventional notion of characteristic lengths shown by most genomic elements (such as genes, exons, introns, transposable elements, etc.), but above all, it is particularly incompatible with the view of the genome as a mosaic of long homogeneous segments or isochores with a typical size [6,18]. This apparent contradiction was solved by using detrended fluctuation analysis (DFA [34]), which revealed the presence of at least three characteristic scales in human chromosomes: short-scale elements (genes, introns, exons, repeats, etc.), medium-scale lengths (corresponding to the typical isochore size), and very-large-scale genome elements (compositional superstructures [21]).

### 4.1. Detection of Genome Compositional Superstructures by Segmentation

DFA analysis shows that isochores with a median length of 116 Kbp (Table 2) are not the largest compositional structures in the genome. Two independent methods were used to show that isochores are in fact organized into longer compositional structures [24]. On one hand, compositional autocorrelation analysis was employed to examine the G+C content of isochores. This analysis revealed that the G+C content of isochores is not independent between each other but exhibits correlations over very large distances, indicating the existence of the clusters of isochores of similar G+C compositions. On the other hand, DNA walks [15] were used to demonstrate the existence of enormous DNA segments (median size ~6 Mbp, Table 3 and Table 4) with a defined G+C composition and typical sizes that are consistent with the sizes of the isochore clusters obtained via autocorrelation analysis. These segments, called compositional superstructures [24], were obtained by means of a modified segmentation algorithm applied to the DNA sequence that was previously converted into a binary S/W sequence (see Section 2). In brief, this modification consists of changing the criterion to evaluate the significance level. While the regular segmentation considers that a sequence is homogeneous (i.e., it remains unsegmented) when its heterogeneity is similar to that in a random sequence, the modified segmentation algorithm takes into account long-range correlations in the DNA chain. In this way, the model for homogeneous DNA sequence comprises fractional Gaussian noise instead of a pure i.i.d. random sequence [37]. 

This algorithm, when applied to entire chromosome sequences, is capable of systematically detecting the boundaries between the above-mentioned large compositional superstructures using rigorous statistical criteria. The coordinates for the 113 superstructures detected in T2T human chromosome sequences are shown in Table S1. Table 3 shows the statistics of lengths and GC% of human T2T superstructures grouped by chromosome, while Table 4 shows a summary statistic.

### 4.2. Hierarchical Organization of Compositional Genome Structures

It is widely known that the GC content of nucleotide sequences usually varies between different genomic elements or regions within the chromosome: exons vs. introns; early vs. late replication genes; regions rich in short- vs. long-interspersed repeats; CpG-poor vs. CpG-rich regions; G- vs. R-chromosome bands; and between different classes of chromosome territories [59,60], isochores [6], and genome superstructures [24].

The lower lengths of isochores compared to superstructures mean that both compositional structures are imbricated in the chromosome, usually being the first contained within the second ones. Table S1 shows the number, length, and GC% of isochores embedded within each superstructure of the T2T human genome, showing again that superstructures are nothing more than the high-level organization of isochores in the genome [24]. There is a median average of 33 isochores by superstructure, reaching a maximum of 796 isochores within the largest superstructure (>200 Mbp) of human chromosome 1. Note that there also exist some superstructures devoid of isochores, above all in the 5′ and 3′ ends of some chromosomes. 

**Figure 2:**
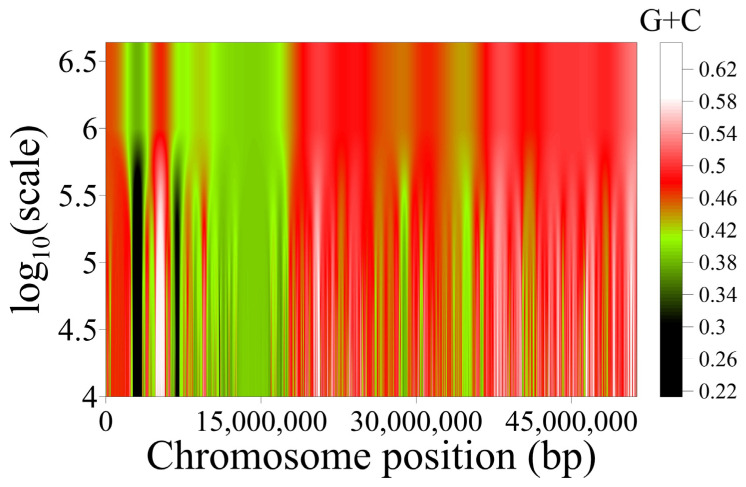
Variation in nucleotide composition along T2T human chromosome 22 at scales between 10 Kbp and 4 Mbp, as revealed by wavelets. The two genome superstructures of this chromosome (green on the left and red on the right) are clearly revealed. The finer-grained isochore structure at lower scales is also discernible.

**Figure 3:**
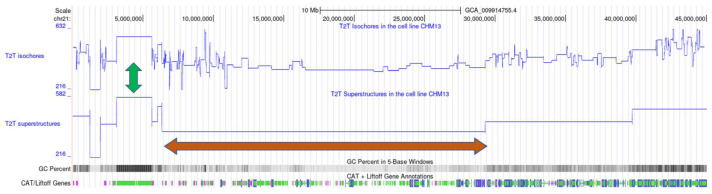
Comparison of isochore and superstructure maps in human T2T chromosome 21 by means of the UCSC Genome Browser. The online image can be observed at the following website: https://genome.ucsc.edu/cgi-bin/hgTracks?db=hub_3267197_GCA_009914755.4&lastVirtModeType=default&lastVirtModeExtraState=&virtModeType=default&virtMode=0&nonVirtPosition=&position=chr21%3A1%2D45090682&hgsid=1583990213_Mq2SxT3AB7CVJP4gui2lAeO3ZljM (accessed on 20 April 2023). The blue lines indicate the GC content of each isochore or superstructure. The orange arrows point to a region of chromosome 21 with low GC content, known as the big ‘gene desert’. The green arrows indicate a region where isochore and superstructure boundaries overlap.

The isochores’ organization within superstructures in the genome is beautifully illustrated by wavelet analysis (Figure 2), as well as by the genome maps of both structures obtained with the help of the UCSC Genome Browser (Figure 3).

The organization of all these interdependent structural compositional components into different length scales, ranging from individual nucleotides to entire chromosomes, leads to a complex compositional structure of the genome. This genome complexity is hierarchically organized, as shown by the existence of domains within domains [36,61] and isochores within genome superstructures [24]. In this way, as it occurs in other levels of biological organization [62], hierarchical complexity is the rule for the compositional structure of the genome.

### 4.3. Functional Significance of Compositional Structures

The significant variations in gene, SINE, and LINE densities [7,63], as well as differences in replication timing [39] and recombination rates [40], all point to the biological meaning of the compartmentalized genome structure. 

On the other hand, the analysis of gene ontology (GO) terms [64] suggests functional significance for the compartmentalization of the genome into both isochores and superstructures as well. Gene pairs embedded in both isochores and superstructures have a higher probability of sharing functional GO terms than random samples of genes, thus pointing to its biological functional relevance [24].

## 5. Segment Compositional Signature (*D_JS_*)

In general terms, the genome signature refers to a given pattern or characteristic associated with DNA sequences that can be used to identify and compare distinct species or individuals. Its main advantage is that it works without the need to perform an alignment. Sometimes, genomic signatures can differentiate single individuals, as in forensic science, that are usually compared to tumor cells in medicine, etc. In comparative genomics and evolutionary biology where the objective is to carry out comparisons among different species, genome signatures are based on statistical properties of DNA sequences that are species-specific; for example, the distribution of *k*-words along the genome is widely used [29].

However, despite the numerous applications of the *k*-word distribution in phylogenetic studies [65,66], as well as in the classification of unknown DNA fragments in metagenome studies [67] or horizontal DNA transfer [68], the main limitation of all genome signature representations based on oligomer frequencies is the lack of divergence among some distantly related species [69]. This effect may be due to the fact that the spatial information retained by distributions of *k*-words is limited to very short scales (actually, *k* nucleotides of distance) and completely ignores the presence of structures at larger scales.

**Figure 4:**
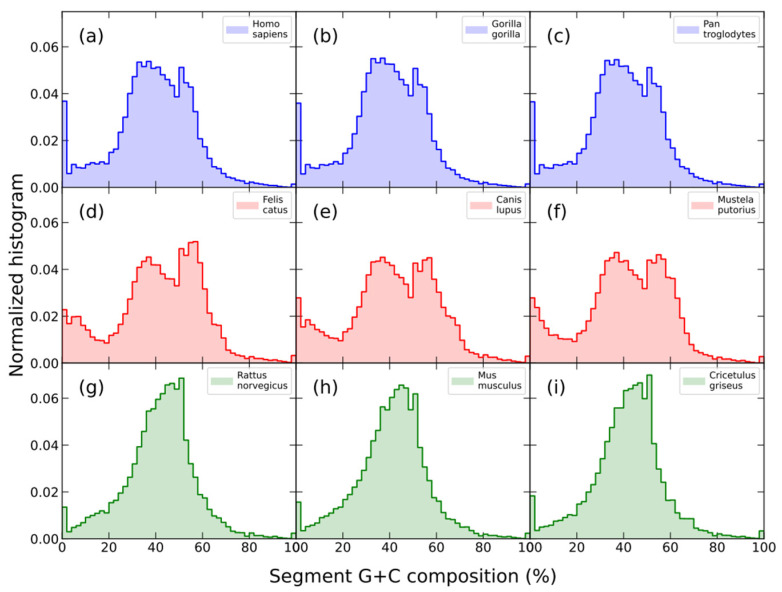
G+C composition histograms of the segments obtained by means of a segmentation algorithm at the *s* = 0.95 significance level of the complete genomes of three primates: human (**a**), gorilla (**b**), and chimpanzee (**c**); three carnivorous: cat (**d**), dog (**e**), and polecat (**f**); and three rodents: rat (**g**), mouse (**h**), and Chinese hamster (**i**). Note that all histograms in the same row, which correspond to closely related species in terms of evolutionary divergence time (http://www.timetree.org (accessed on 20 April 2023)), look quite similar to each other.

We briefly reviewed a new signature based on compositional genome structure called *segment compositional signature*, which has been recently proposed [70]; it is defined as the distribution of the G+C composition of DNA segments obtained by means of the segmentation algorithm defined above [35,36,71]. We have observed that the histograms of the G+C composition for segments are similar for closely related species, while histograms for distantly related species show different patterns (Figure 4).

To quantify the difference between the two species [70], we use the square root of the Jensen–Shannon divergence between their histograms (*D_JS_*). This measure is known to have all properties of a distance in the mathematical sense [72]; thus, it is a good candidate for a molecular sequence metric [73], i.e., a measure of the “distance” between DNA sequences.

A good correlation was observed between the distance, *D_JS_*, between *Homo sapiens* and a set of mammals for which a complete genome is available and the evolutionary divergence time (time from the common ancestor) [70].

## 6. Sequence Compositional Complexity (SCC)

Once a sequence is segmented into an array of *m* homogeneous compositional domains $\left\{S_{1}S_{2},\ldots ,S_{m}\right\}$, a reliable measure of sequence compositional complexity, or SCC [25], can be computed using the generalization of the Jensen–Shannon divergence relative to *m* sequences:

(3) $$SCC=H\left(S\right)-\sum_{i=1}^{m}\frac{n_{i}}{N}H\left(S_{i}\right)$$

where *S* denotes the entire genome sequence, *N* denotes its length, *n_i_* denotes the length of the *i*th domain *S_i_*, and $H\left(\cdot \right)$ denotes again the Shannon entropy of the distribution of the relative frequencies of symbol occurrences, which are denoted by $\left\{f_{A},f_{T},f_{C},f_{G}\right\}$ in the corresponding (sub)sequence (Equation (2)). It should be noted that the above expression is the same as the one used in the segmentation process, and it is applied to the two tentative new subsequences (*m* = 2) to be obtained in each step. Thus, the two steps of the SCC computation are based on the same theoretical background.

SCC has particularly convenient properties for measuring compositional genome structure complexity:

1. The SCC value is 0 if no segments are identified in the sequence, indicating that it is compositionally homogeneous, such as a random sequence.
2. By using a statistical significance threshold over the segmentation step, SCC ensures that the difference between each pair of adjacent domains is not merely due to statistical fluctuations.
3. SCC has a high sensitivity to sequence changes. A single nucleotide substitution, or a small indel, can often be sufficient to alter the number, length, or nucleotide frequencies of compositional domains and, consequently, affect the resulting SCC value.
4. It increases/decreases with both the number of segments and the degree of compositional differences among them. In this way, SCC is analogous to the measure used by McShea and Brandon [74] for obtaining complexity estimates based on morphological characters: an organism is more complex if it has a greater number of parts and/or a higher differentiation among these parts.
5. It is based on analyzing the underlying distribution of segment lengths and nucleotide compositions at various levels of detail [37], thus fulfilling one of the key requirements for a reliable complexity measure [28].

## 7. Phylogenetic Trends of Compositional Genome Structure

Phylogenetic trends (evolutionary changes arising within a group of organisms over time) are usually found for morphological, anatomical, physiological, or biochemical traits, which allow establishing whether the selection is acting on a given trait. The compositional structure of genomes is a complex trait that, when measured by SCC, enables the detection of increasing or decreasing phylogenetic trends, thereby revealing the influence of positive or negative selection pressure on the compositional structure of entire genomes.

We review here the evolutionary trends towards higher SCC that were recently found in some species of the ancient Cyanobacteria phylum [3]. These microbes were essential for the development of life on Earth. According to the fossil record, the phylum’s origins occurred more than 2.5 billion years ago (Bya) [75,76]. Cyanobacteria altered the course of life on Earth by re-releasing oxygen via photosynthesis, which resulted in the Great Oxidation Event about 2.3 billion years ago [77]. This critical event allowed for the emergence of complex multicellular life forms [78].

Using ridge regression of SCC against time on a maximum likelihood phylogenomic tree of ninety-one cyanobacterial genomes, positive trends towards higher genome complexity in more evolved clades with long-branch distances with respect to the root of the tree were found [3]. Furthermore, three standard tests to distinguish passive vs. driven progressive evolution provide evidence for the progressive evolution of SCC driven by natural selection.

## 8. Conclusions

DNA structural biology attempts to understand the three-dimensional structure of DNA, which can determine its cellular function. While the DNA sequence composition determines key structural properties of the DNA fiber, such as its stability, flexibility, or susceptibility to damage, the chart maps of DNA-binding proteins and related chromatin structures show the interplay between chromatin and genome function. Both approaches can jointly reveal emerging roles for large-scale compositional domains and higher-ordered chromatin organization.

The analytical ultracentrifugation of bulk DNA and application of statistical physics methods (power spectra, fluctuation analysis in DNA walks, and entropic segmentation) on the first long DNA sequences were able to reveal a large-scale compositional, power law genome structure formed by isochores (of a typical size of ~300 Kbp) and larger superstructures (around several Mbp). The application of these statistical physics methods to the recently obtained first complete 3.055 billion base pair sequence of a human genome provides an unprecedented opportunity to validate both compositional models. To this end, we shared a database with the genome coordinates of both isochores and superstructures in the T2T sequence, as well as an interactive genome map, thus allowing interested researchers to retrieve data to test specific hypotheses about genome structure.

Short-scale structures for genome elements, long-scale structures for isochores, and very-long-scale structures for superstructures are all imbricated and hierarchically organized in the chromosome, as shown by the existence of domains within domains and isochores within genome superstructures. Thus, similarly to what occurs at other levels of biological organization, the genome exhibits a hierarchical compositional structure. Once such a compositional structure is determined, a measure of its sequence compositional complexity (SCC) can be derived, which can be also used for comparisons between genomes. Finally, as a case study, we reviewed the positive evolutionary trends towards higher SCC that were recently found in species of the ancient Cyanobacteria phylum, which provided the first evidence for the driven progressive evolution of genome compositional structure.

The availability of complete T2T genomes [13] in an increasing number of species, together with pangenome projects [79], which capture known variants and haplotypes and reveal new alleles at structurally complex loci, will hopefully soon provide DNA sequences of sufficient length and quality to allow a further, robust validation of specific hypotheses on genome structure.

## Figures and Tables

**Table 1:** Lengths and GC% of T2T human isochores by chromosome.

		Length			GC%		
Chromosome	*N*	Min	Median	Max	Min	Median	Max
chr1	1113	30,004	102,983	5,403,580	32.80	43.02	67.96
chr2	897	30,004	128,804	4,304,270	31.57	41.20	66.39
chr3	656	30,004	127,485	5,001,190	21.54	41.23	62.17
chr4	427	30,004	234,223	5,642,550	23.80	39.15	72.64
chr5	561	30,004	164,800	7,206,270	30.21	40.70	62.46
chr6	510	30,004	163,262	3,500,380	32.19	40.85	58.39
chr7	596	30,004	119,830	3,412,220	33.19	42.44	68.05
chr8	449	30,004	147,916	4,875,420	33.27	41.36	63.98
chr9	501	30,004	107,885	22,256,800	31.74	42.57	65.87
chr10	562	30,004	121,504	3,195,210	32.63	42.09	72.51
chr11	551	30,004	109,143	3,008,680	33.64	42.81	62.56
chr12	505	30,005	115,060	3,649,550	32.99	42.49	63.91
chr13	317	30,004	128,955	10,449,500	21.22	40.27	60.57
chr14	426	30,004	101,284	3,881,480	21.89	42.01	63.58
chr15	461	30,004	104,795	7,482,370	21.37	42.62	62.03
chr16	457	30,004	82,508	12,645,100	33.24	44.86	66.31
chr17	517	30,004	82,785	4,713,850	33.08	45.61	62.57
chr18	237	30,004	180,918	3,584,850	34.03	40.14	56.08
chr19	313	30,004	101,006	2,676,290	35.20	48.09	65.30
chr20	314	30,004	101,682	2,232,290	32.89	44.17	65.15
chr21	178	30,004	95,755	4,852,870	21.63	42.14	63.20
chr22	342	30,004	68,700	1,690,830	21.25	46.26	64.72
chrX	366	30,004	166,946	14,835,700	22.41	40.66	62.24

**Table 2:** Basic length and GC% statistics in T2T human isochores.

	N	Minimum	Median	Maximum
Length (bp)	11,256	30,005.00	116,447.00	22,256,800.00
GC%	11,256	21.22	42.24	72.64

**Table 3:** Length and GC% statistics of human T2T genome superstructures by chromosome.

		Length			GC%		
Chromosome	*N*	Min	Median	Max	Min	Median	Max
chr1	4	328,708.00	23,816,031.50	200,426,557.00	40.02	45.53	58.20
chr2	4	3,360,333.00	51,107,636.50	137,121,146.00	38.23	43.43	50.92
chr3	3	36,078,355.00	73,698,202.00	91,329,391.00	35.28	39.59	41.28
chr4	4	489,000.00	4,883,872.00	183,318,201.00	37.50	44.89	55.27
chr5	6	1,719,665.00	10,580,851.50	128,874,195.00	38.12	44.70	53.09
chr6	5	4,367,830.00	18,410,867.00	104,707,592.00	37.67	41.22	46.35
chr7	4	2,840,530.00	7,380,668.00	142,965,562.00	39.85	46.63	54.87
chr8	5	532,709.00	18,506,340.00	74,252,182.00	38.17	41.71	55.45
chr9	5	745,901.00	7,358,207.00	105,799,637.00	37.88	48.44	56.06
chr10	6	31,699.00	790,594.50	122,866,079.00	41.00	45.76	72.51
chr11	3	230,004.00	3,096,068.00	131,801,697.00	41.22	42.54	55.51
chr12	5	295,312.00	8,431,710.00	98,848,568.00	39.05	45.18	52.94
chr13	7	167,828.00	3,576,422.00	99,522,852.00	21.87	42.77	57.94
chr14	6	935,799.00	5,282,438.00	78,687,833.00	39.51	45.26	55.98
chr15	1	99,753,195.00	99,753,195.00	99,753,195.00	42.12	42.12	42.12
chr16	8	2,323,621.00	6,794,467.50	37,570,616.00	36.35	46.71	57.98
chr17	3	10,136,275.00	22,311,188.00	51,829,434.00	41.51	45.56	52.19
chr18	6	299,493.00	2,825,872.00	64,251,224.00	35.92	42.88	53.05
chr19	7	33,056.00	4,915,345.00	30,358,228.00	35.71	47.91	56.35
chr20	4	2,912,961.00	16,593,634.00	30,110,026.00	40.53	45.21	55.82
chr21	9	307,155.00	1,240,965.00	22,967,603.00	21.63	43.15	58.18
chr22	2	17,629,880.00	25,662,463.00	33,695,046.00	41.31	44.77	48.23
chrX	6	272,109.00	1,306,918.50	148,323,701.00	39.21	46.71	55.04

**Table 4:** Summary statistics of length and GC% in human T2T genome superstructures.

	*N*	Minimum	Median	Maximum
Length (bp)	113	31,699.00	6,111,300.50	200,427,000.00
GC%	113	21.63	45.10	72.51

## Data Availability

Not applicable.

## Supplementary Materials

The following supporting information can be downloaded at https://www.mdpi.com/article/10.3390/biology12060849/s1. Table S1: Superstructure coordinates and the number, length (bp), and GC% of the isochores embedded within superstructures in T2T human chromosomes.
